# Abdominal Computed Tomography with a Twist: The ‘Whirl Sign’ for Mesenteric Volvulus

**DOI:** 10.5811/cpcem.2020.3.46682

**Published:** 2020-05-18

**Authors:** Jodi Spangler, Jonathan Ilgen

**Affiliations:** University of Washington, School of Medicine, Department of Emergency Medicine, Seattle, Washington

**Keywords:** Volvulus, whirl sign, bowel obstruction

## Abstract

**Case Presentation:**

A 55-year-old woman with a history of end-stage renal disease, peripheral vascular disease, and multiple prior abdominal surgeries presented to the emergency department with three days of diffuse, severe, abdominal pain with accompanying nausea, emesis, and food intolerance. A computed tomography (CT) of her abdomen demonstrated a “whirl” of small bowel and mesenteric vessels, raising suspicion for mesenteric volvulus and resultant small bowel obstruction.

**Discussion:**

Mesenteric volvulus is a low incidence, high mortality condition; therefore, early recognition and operative intervention are critical. Patients with a “whirl sign” on CT are more likely to require surgical intervention for their small bowel obstruction.

## CASE PRESENTATION

A 55-year-old woman with a history of end-stage renal disease, peripheral vascular disease, and multiple prior abdominal surgeries presented to the emergency department with three days of diffuse, severe, abdominal pain with accompanying nausea, emesis, and food intolerance. Her physical examination was remarkable for a soft, slightly distended abdomen with diffuse tenderness to palpation. She had no guarding or rebound. A computed tomography (CT) of her abdomen demonstrated a “whirl” of small bowel and mesenteric vessels (Video), raising suspicion for mesenteric volvulus and resultant small bowel obstruction.^1^,^2^

In this patient, an exploratory laparotomy was performed amid concern for small bowel ischemia, and a mesenteric volvulus was confirmed intraoperatively. A small bowel resection with extensive adhesiolysis was performed, and multiple mesenteric lymph nodes were excised. The patient had an unremarkable postoperative course and was discharged home.

## DISCUSSION

**Image 1:**
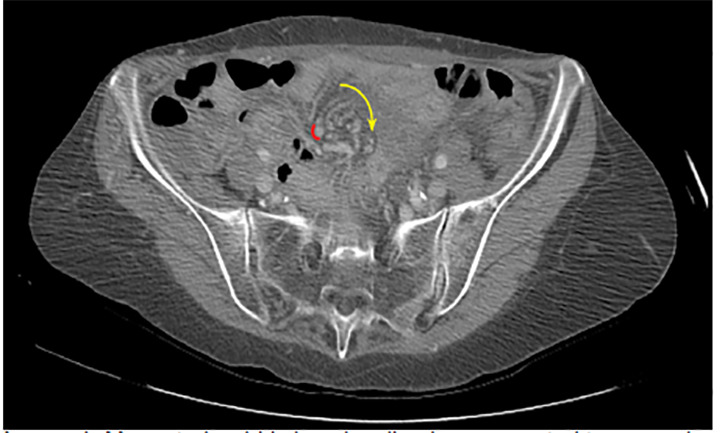
Mesenteric whirl sign visualized on computed tomography (yellow arrow).

**Image 2:**
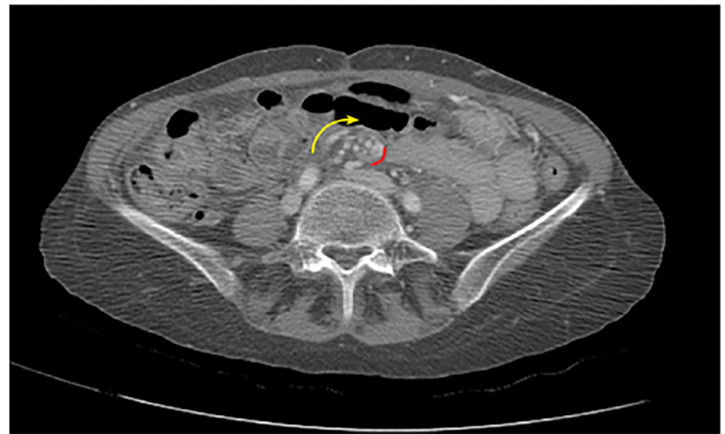
Still images: Sequential computed tomography images demonstrate small bowel and mesenteric vessels rotating in mass with soft tissue and fat attenuation. Hash mark highlights the position of a specific piece of mesentery as it revolves; arrow indicates movement of the mass.

Mesenteric volvuli occur when bowel twists around its mesenteric root.^1^ This results in bowel wall and vascular compression, with subsequent intestinal obstruction and ischemia. Mesenteric volvuli are classified as “primary” when occurring in the setting of an otherwise normal abdominal cavity, and “secondary” when occurring in the setting of pre-existing lesions such as adhesions or malrotation.^3^ Abdominal pain is the typical presenting symptom, and despite its low incidence, mortality rates from mesenteric volvuli are high; thus, early recognition and operative intervention are critical.^3^ The “whirl sign” on CT imaging (Images 1 and 2) is a highly specific finding for intestinal volvulus (albeit poorly sensitive), and should raise suspicion for a closed loop obstruction.^4^ Presence of the whirl sign is helpful for guiding management of patients with clinical and radiologic signs of small bowel obstruction, as patients with this finding are 25 times more likely to require surgery than those without this finding on imaging.^4^

**CPC-EM Capsule**

What do we already know about this clinical entity?

Mesenteric volvulus occurs when bowels twists around its mesenteric root and can result in bowel wall and vascular compression, intestinal obstruction and ischemia.

What is the major impact of the image(s)?

Mesenteric volvulus is a low incidence, high mortality condition. Therefore, early recognition and operative intervention are critical.

How might this improve emergency medicine practice?

Whirl sign is helpful for guiding management of patients with signs of small bowel obstruction, as patients with this finding are more likely to require surgery.

## Supplementary Information

Video Mesenteric whirl sign. Computed tomography of the abdomen demonstrates a swirling mass (see arrow) of soft-tissue and fat attenuation indicative of twisted loops of small bowel and mesenteric vessels.
